# COVID-19 and Subsequent Development of Pancytopenia With Concurrent Disseminated Intravascular Coagulation

**DOI:** 10.7759/cureus.32845

**Published:** 2022-12-22

**Authors:** Michael D Dube, Sumina Sapkota, Khushboo Lakhatariya, Anthony Russo

**Affiliations:** 1 College of Medicine, Northeast Ohio Medical University, Rootstown, USA; 2 Department of Internal Medicine, Trumbull Regional Medical Center, Warren, USA

**Keywords:** anemia, itp, dic, pancytopenia, covid-19

## Abstract

Complications resulting from coronavirus disease 2019 (COVID-19) sequelae have been well documented. These include blood conditions such as lymphopenia, thrombocytopenia, and hypercoagulability. Less common problems that may arise are disseminated intravascular coagulation (DIC), immune thrombocytopenic purpura (ITP), and pancytopenia. Furthermore, the majority of COVID-19 patients to develop pancytopenia have been immunosuppressed. We present a case of a previously immunocompetent patient who subsequently developed pancytopenia, DIC, as well as symptoms of ITP one month after being diagnosed with COVID-19.

## Introduction

Hematologic disorders reported being associated with coronavirus disease 2019 (COVID-19) include lymphopenia, neutrophilia, and thrombocytopenia as well as arterial and venous thromboses [1]. Patient presentations consistent with disseminated intravascular coagulation (DIC) and immune thrombocytopenic purpura (ITP) have also been reported [2,3]. Furthermore, pancytopenia is a known but rare manifestation of COVID-19, with most instances occurring in immunocompromised patients [4,5]. However, there has only been a limited number of cases of pancytopenia occurring in the presence of COVID-19 in an immunocompetent patient [6,7]. We present a novel case of an immunocompetent patient who not only developed DIC but also pancytopenia leading to multi-organ failure.

## Case presentation

A 71-year-old female unvaccinated against COVID-19 with a past medical history of hyperlipidemia, hypertension, hypothyroidism, kidney stone, stage III chronic kidney disease, and esophageal stricture presented to the emergency department (ED) with weakness, nausea, vomiting, and diarrhea since being diagnosed with COVID-19 a month prior. The morning before her ED visit, she developed a sore throat and some difficulty swallowing, which made her eating and drinking more difficult. She did have some difficulty ambulating due to weakness as well as shortness of breath on exertion, and cough productive of yellow sputum for the past six days. She denied fever, chest pain, hematemesis, melena, hematochezia, or abdominal pain.

**Figure 1:**
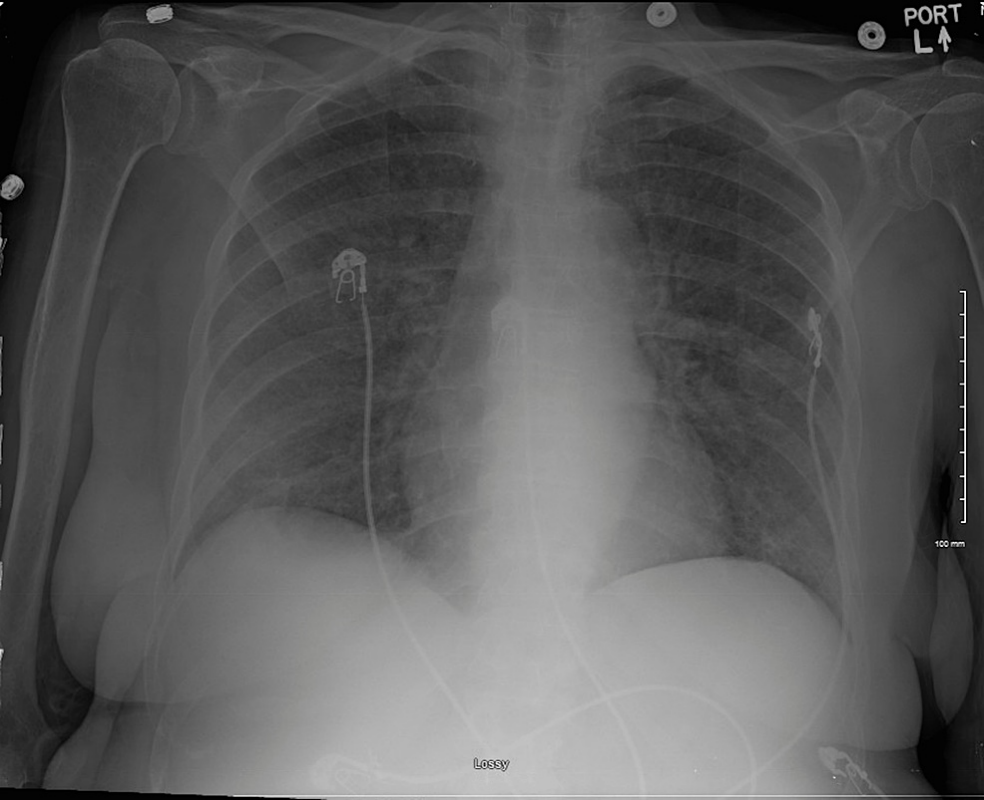
Chest x-ray upon presentation to the emergency department

On initial vitals, the patient was afebrile with a temperature of 98.3 degrees Fahrenheit, heart rate of 89 beats per minute, respiratory rate of 18 breaths per minute, blood pressure of 117/66 mmHg, and oxygen saturation of 95% on room air. The patient’s laboratory studies showed pancytopenia (white blood cell count of 3.5 × 10^3^ cells/μL with left shift, hemoglobin of 11.6 g/dL, platelet count of 42 × 10^3^ cells/μL), acute-on-chronic kidney injury (creatinine of 2.89 mg/dL), slightly elevated aspartate transaminase of 39 U/L with normal alanine transaminase, normal bilirubin, normal alkaline phosphatase, and hyperammonemia (78 mcmol/L). Chest x-ray was obtained in the ED, which showed diffuse interstitial airspace disease (Figure 1). Procalcitonin was elevated at 1.13 ng/mL. She was given IV fluid and levofloxacin and admitted to the floor for presumed pneumonia, acute-on-chronic renal failure, and generalized weakness. 

The patient’s pancytopenia continued to worsen with a white blood cell count of 1.8 × 10^3^ cells/μL, Hbg of 9.7 g/dL, and platelets of 26 × 10^3^ cells/μL on day 2 of hospital admission. The patient did have a slightly nodular contour of the liver noted on abdominal CT with borderline splenomegaly; however, she denied any alcohol use and had a negative alpha 1 antitrypsin level as well as a non-reactive hepatitis B surface antibody. Fibrinogen level was measured, which was 119 mg/dL on admission day 3 and a d-dimer the following day was elevated at 4.29 ug/ml fibrinogen equivalent units (FEU) with a prothrombin time (PT) of 14 seconds, international normalized ratio (INR) of 1.3, and partial thromboplastin time (PTT) of 13.5 seconds. The patient’s platelets dropped as low as 13 × 10^3^ cells/μL on day 7 even after a total of four platelet transfusions. Intravenous Immunoglobulin (IVIG) was started on admission day 8 for presumed ITP with no resolution of pancytopenia. The patient’s ammonia rose to 111 mcmol/L on admission day 10 while on rifaximin and lactulose and was transferred to the intensive care unit (ICU) secondary to altered mental status (AMS) with a Glasgow Coma Scale (GCS) score of 7/15 and transfused platelets again. A summary of the patient's notable laboratory values throughout the hospital stay is provided in Table 1.

**Table 1:** Laboratory analyses AST: aspartate transaminase; ED: Emergency Department; WBC: white blood cells; PT: prothrombin time; PTT: partial thromboplastin time; INR: international normalized ratio

Variable	On Admission	Day 2	Day 3	Day 7	Day 10
WBC × 10^3^ cells/μL	3.5	1.8	-	-	-
Hemoglobin g/dL	11.6	9.7	-	-	-
Platelet count × 10^3^ cells/μL	42	26	-	13	-
Creatinine mg/dL	2.89	-	-	-	-
AST U/L	39	-	-	-	-
Ammonia mcmol/L	78	-	-	-	111
Fibrinogen mg/dL	-	-	119	-	-
D-dimer ug/mlFEU	-	-	4.29	-	-
PT s/INR	-	-	14/1.3	-	-
PTT s	-	-	13.5	-	-

**Figure 2:**
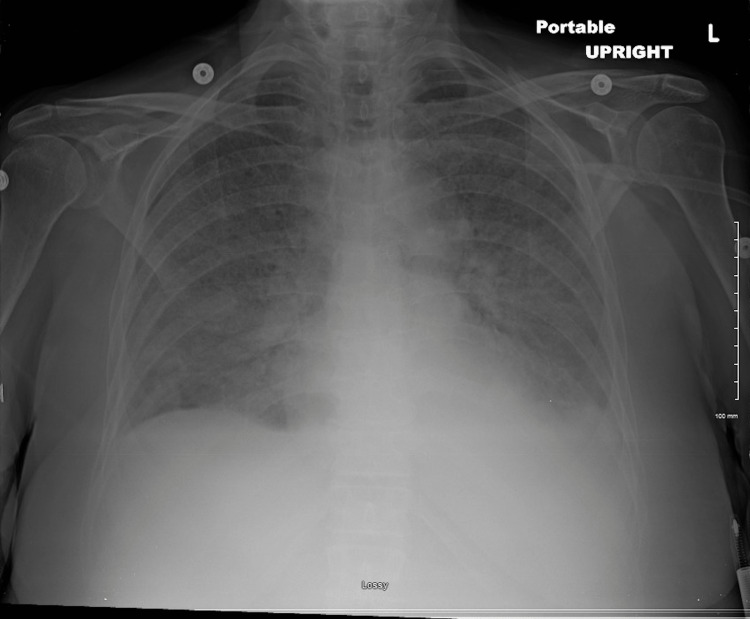
Chest x-ray on admission day 8

The patient was also in acute hypoxic respiratory failure and intubated after increasing oxygen requirement of up to 6L on the floor with worsening diffuse interstitial airspace disease on admission day 8 (Figure 2). The patient’s peripheral blood smear showed rare schistocytes, a good number of young white blood cells, and nucleated red blood cells. Hemolysis was deemed less likely and bone marrow disorder could not be ruled out. After finishing a course of IVIG, the patient still did not improve. While in the ICU, the patient was found to have a Sequential Organ Failure Assessment (SOFA) score of 16, was changed to a code status of Do not resuscitate comfort care arrest (DNRCCA), extubated, and passed away on admission day 11.

## Discussion

It has previously been hypothesized that COVID-19 causes pancytopenia by autoantibody targeted destruction of blood cells [8]. Severe acute respiratory syndrome coronavirus 2 targets angiotensin-converting enzyme 2 receptors, which have been found in the bone marrow and also have an impact on the fibrinolysis system [8,9]. Therefore, binding to these receptors can cause downstream effects including pancytopenia, ITP, and DIC [10]. Furthermore, there are various case reports that have hypothesized the role of proinflammatory cytokines in the impairment of hematopoiesis [8]. Additional studies are needed to determine the exact mechanism that causes specific hematological complications.

Hematological problems have been reported in association with COVID-19 including anemia, DIC, and ITP [1]. DIC is a well-known complication of infected patients, although the majority of patients present with only mildly decreased platelet count unlike the dramatic drop seen in our patient [2]. Due to this, ITP was suspected; however, the patient did not improve after IVIG treatment like in previously reported studies [11,12]. Pancytopenia is another known but rare complication of COVID-19 [8]. Yet, most cases have been described in immunosuppressed patients [4,5]. Bridwell et al. did report the occurrence of pancytopenia in an otherwise healthy 40-year-old male who was found to be COVID-19 positive upon presentation [13]. Conversely, our patient was diagnosed with COVID-19 a month prior to presentation and had laboratory studies consistent with DIC. The patient did have a negative COVID-19 polymerase chain reaction (PCR) test upon admission to the hospital but the constellation of symptoms with worsening pulmonary disease was more likely secondary to COVID-19 sequelae from a previous infection. Barranco-Trabi et al. also confirmed a case of pancytopenia in a COVID-19-positive patient; however, the presentation was due to underlying pernicious anemia [7]. Although our patient’s presentation is likely multifactorial and not solely due to COVID-19; the development of DIC and pancytopenia, in this case, serves as an example of hematological issues to keep in mind when treating a patient with previously diagnosed COVID-19.

## Conclusions

With the continuously changing nature of COVID-19, it is important to keep in mind the various hematological complications that may arise. Close monitoring and follow-up in the weeks after diagnosis is crucial in preventing further complications. More research is needed to determine the exact mechanism and risk factors for pancytopenia in patients diagnosed with COVID-19.
